# Introducing the good clinical proton practice (GCPP) initiative for enhancing proton therapy standards

**DOI:** 10.1002/acm2.14436

**Published:** 2024-06-18

**Authors:** Robabeh Rahimi, Frank Emert, the PTCOG Treatment Efficiency Subcommittee

**Affiliations:** ^1^ Department of Radiation Oncology University of Maryland School of Medicine Baltimore Maryland USA; ^2^ Center for Proton Therapy Paul Scherrer Institute Villigen Switzerland

Dear Editor

With the expansion of proton therapy, made possible by advances such as compact beam delivery systems, sophisticated treatment planning concepts and innovative patient positioning, access to this cutting‐edge treatment has increased significantly. While this growth is undoubtedly beneficial to a greater number of cancer patients, it also comes with unique challenges, especially for smaller or newly established proton centers. These centers often lack the extensive resources, established workflows and experienced staff found in larger facilities. This is where our initiative comes into play.

## INTRODUCING THE GOOD CLINICAL PROTON PRACTICE (GCPP) INITIATIVE

To address these challenges, we created the GCPP to support all proton therapy centers in their mutual interest, regardless of their size, experience, or resources. The goal of the GCPP is to provide optimized, practical guidelines, technical documentation, and white papers that are essential for daily clinical operations to ensure that every patient receives the highest standard of treatment quality in proton therapy.

## DEFINITION AND SELF‐PERCEPTION OF GCPP

GCPP can be defined as a set of practices motivated by Good Clinical Practice (GCP), an internationally recognized framework of standards in the field of clinical trials applied inter alia in radiotherapy, without focusing on ethics guidelines. Applied to the field of proton and particle therapy, this means that GCPP is based exclusively on scientific criteria designed to guarantee safe, effective, and efficient treatment for patients. In doing so, GCPP focuses on comprehensive quality assurance, standardized operating procedures and established irradiation conditions, paying particular attention to the contribution of medical physics experts.

## KEY FEATURES OF THE GCPP (INSPIRED BY GCP)

1. **Consistent or comparable quality standards**: Just as clinical trials are designed to provide scientifically valid and comparable results regardless of study site by applying the rigorous technical GCP criteria, GCPP aims to focus on practicable and interoperable medical physics practice standards in clinical applications of proton therapy that ensure a comparable high‐quality level of treatment for every patient.
2. **Standardized operating procedures** (SOPs): The greatest possible consistency or comparability of processes is critical to reducing variability in patient treatment and expanding the reproducibility of clinical outcomes.
3. **Involvement of medical physics experts**: Interdisciplinary collaboration is the only way to ensure that the complex medical physics and technical aspects of proton therapy are appropriately translated into clinically successful patient treatments while maintaining the highest scientific standards.

## THE CORE ASPECTS OF THE GCPP INCLUDE

1. **Distribution of resources**: GCPP aims to focus on making medical physics knowledge that is clinically relevant, easily accessible through concise, application‐oriented resources, without focusing on extensive theoretical discourses or complex guidelines.
2. **Sharing knowledge**: By promoting an efficient flow of information between clinical centers, GCPP aims to contribute to the effective dissemination of relevant knowledge, best practices, and innovations. This collaborative approach will enable the sharing of experiences and solutions to common challenges in proton therapy and enable quality of care regardless of the proton center's mission or stage of development.
3. **Engagement and feedback**: Through initiatives such as questionnaires that accompany our guidelines, GCPP aims to actively solicit feedback from the community to ensure that resources are practical and based on real‐world applications.
4. **The nature of GCPP publications**: GCPP publications aim to be concise, practical, and directly applicable to clinical workflows, making them an ideal reference tool for everyday practice. This intended practical focus, with the active solicitation of feedback from the community, will ensure that GCPP resources remain relevant and useful to all proton therapy centers.
5. **Address standardization challenges**: GCPP aims to focus on supporting the current challenges associated with broader applications of proton and particle therapy treatments by promoting adherence to GCP scientific standards within and between proton centers. At the same time, this should create the conditions for successful clinical proton therapy trials to establish this innovative radiation method.

## CONCLUSION

By promoting standardized, high‐quality proton therapy through the GCPP initiative, we aim to ensure that this sophisticated treatment modality could serve its full potential in cancer treatment. We invite the community to participate in GCPP and support the initiative to contribute to the collective effort to combine scientific rigor with practical application, which could serve to ensure long‐term positive patient outcomes in proton therapy.

## CALL AND INQUIRY

The Journal of Applied Clinical Medical Physics (JACMP) seems to us to be the ideal platform for realizing our goals regarding distribution of important resources due to its large readership among clinical medical physicists and its focus on practical, clinically oriented publications. Because of its commitment to advancing the field through accessible and applicable research, the JACMP seems to us to be particularly well suited for the dissemination of the GCPP projects.

## CONFLICT OF INTEREST STATEMENT

The authors declare no conflicts of interest.

